# Sensor Fusion-Based Teleoperation Control of Anthropomorphic Robotic Arm

**DOI:** 10.3390/biomimetics8020169

**Published:** 2023-04-20

**Authors:** Xiaolong Yang, Furong Chen, Feilong Wang, Long Zheng, Shukun Wang, Wen Qi, Hang Su

**Affiliations:** 1 Key Laboratory of Bionic Engineering, Ministry of Education, Jilin University, Changchun 130022, China; 2 College of Mechanical and Electrical Engineering, Changchun University of Science and Technology, Changchun 130022, China; 3 Weihai Institute for Bionics, Jilin University, Weihai 264402, China; 4 School of Future Technology, South China University of Technology, Guangzhou 511436, China; 5 Department of Electronics, Information and Bioengineering, Politecnico di Milano, Milan 20133, Italy

**Keywords:** sensor fusion, anthropomorphic robotic arm, teleoperation control

## Abstract

Sensor fusion is a technique that combines information from multiple sensors in order to improve the accuracy and reliability of the data being collected. In the context of teleoperation control of an anthropomorphic robotic arm, sensor fusion technology can be used to enhance the precise control of anthropomorphic robotic arms by combining data from multiple sensors, such as cameras, data gloves, force sensors, etc. By fusing and processing this sensing information, it can enable real-time control of anthropomorphic robotic arms and dexterous hands, replicating the motion of human manipulators. In this paper, we present a sensor fusion-based teleoperation control system for the anthropomorphic robotic arm and dexterous hand, which utilizes a filter to fuse data from multiple sensors in real-time. As such, the real-time perceived human arms motion posture information is analyzed and processed, and wireless communication is used to intelligently and flexibly control the anthropomorphic robotic arm and dexterous hand. Finally, the user is able to manage the anthropomorphic operation function in a stable and reliable manner. We also discussed the implementation and experimental evaluation of the system, showing that it is able to achieve improved performance and stability compared to traditional teleoperation control methods.

## 1. Introduction

The development of anthropomorphic robotic arms has received more and more attention in the robot industry in recent years [1]. By analyzing the human arm skeleton, joint movements and muscle forces in depth, the anthropomorphic robotic arm aims to develop a human-like mechanical structure, motion planning and control theory [2,3]. The practice has proved that anthropomorphic robotic arms can assist or even replace humans to complete auxiliary and heavy tasks in multiple fields, such as industry and biomedical and social services [4,5]. In particular, the anthropomorphic robotic arms with humanized control, such as Yumi robotic arm, Robonaut2 robotic arm and Justin robotic arm, can be more flexible and coordinated.

Traditional control of anthropomorphic robotic arms suffers from various limitations. Direct use of control instructions to control robot action is a complex and mechanized operation, easily causing operator fatigue and low efficiency [6], and requires prior training of the operator. Although the human–computer interaction mode of the graphical interface improves the quality of interaction, it still cannot remove the command input of the mouse and keyboard [7,8], and the efficiency improvement is not obvious. Additionally, the manual controller provides more limited space for the operator.

Teleoperation control based on body sensors has the characteristics of stability and flexibility, which can better simulate human movement and meet the needs of contactless service under epidemic conditions [9,10,11,12]. Among the many signal sources available, the motion of the arm is an accurate and stable signal source for hand tracking and arm movement [13]. Currently, arm-based teleoperated devices can be divided into wearable and non-wearable types [14]. Wearable sensors, for example, Myo arm rings and body gloves [15], take the sensor (inertial sensor, bending sensor) signal as the input signal to detect the EMG signal of the external surface of the hand [16,17], which can quickly and accurately receive the arm movement information and is not affected by light, occlusion and other factors. However, wearing the sensor for a long time can cause discomfort and cumbersome operations that require calibration before use [18]. Non-wearable sensors are mainly depth cameras, such as Kinect, Leap Motion and other binocular cameras, which use arm images as input signals to identify the joints of the hand and thus calculate the posture information of the human arm without the need for wearable devices [19], which is more flexible and convenient, but susceptible to the effects of occlusion and lighting conditions, and requires computationally heavy and complex algorithms [20].

Because of the limited single-mode equipment, the arm tracking is not complete [21], making the complex control application impossible. Only within 60° of palm rotation can the leap motion sensor (horizontal field of view angle 140°, vertical field of view angle 120°, interaction depth 10–60 cm) fully recognize the hand. When the palm rotation is at 90 degrees or even at a rotation of 180 degrees, there is finger occlusion, and it is unable to identify the correct movement of the fingers [22,23]. Vision-based robot control, especially visual servo-control algorithm research results [24] are numerous [25]. Andrzej Milecki calibrated the vision system, and then recognized the position of three markers assembled on the operator’s hand and arm, and used it for the control system of an electro-hydraulic servo-driven two-axis manipulator to track the human hand [26]. Y. Benezeth proposed a vision-based static camera indoor human detection and tracking system, which improves the performance of the entire system and reduces the complexity of the task [27].

Some researchers combine different teleoperation device [28] modes to improve arm tracking accuracy and stability. The experimental results of multimodal fusion, for example, Myo Armband and Leap Motion camera fusion [23,29], Kinect and EMG Fusion [30], Leap motion and data glove fusion [31,32], Vision and EEG [33], etc., have effectively verified the complementary advantages of multi-modal information.

Conventional robots are mainly controlled based on single-modal signals, and their control robustness is poor and vulnerable to the external environment. However, dual-arm robot control based on multi-modal signal sensing has not only greatly improved the flexibility and accuracy of the dual-arm robot control under unknown environmental conditions, but also ensured that the dual-arm robots work more reliably in the process of cooperative operation. In this paper, we designed a dual-arm tracking control system based on multimodal signal perception, fusing the signals from the Kinect vision sensor, data glove and force sensor by using the algorithm, used it to control the anthropomorphic robotic arm and dexterous hand in real-time, and then verified the effectiveness and stability of multimodal signal perception and control of data:

- Multimodal sensor fusion control overcomes the inherent defect of single mode and is more flexible and applicable to the operator.
- Multimodal sensor fusion control conforms to the characteristics of bionics in humanoid motion to improve the coordination and naturalness of dual-arm robot control.
- Multimodal sensor fusion teleoperation control has higher efficiency and can be carried out much more smoothly.

In general, one arm of the human body has a scapula, a clavicle, a humerus, a radius, an ulna, 8 wrist bones, 5 metacarpals and 14 phalanges. The shoulder joint corresponds to a spherical sub (rotation about X, Y and Z axes) with three degrees of freedom; the elbow joint corresponds to a rotational sub (rotation about X axis) with one degree of freedom; the wrist joint has two directions of rotation (rotation about X and Z axes) with two degrees of freedom; the ulna and flexor bones, which connect the wrist joint to the elbow joint, have a cross-rotational motion between them (rotation about Y axis) with one degree of freedom.

## 2. Methodology

### 2.1. Mechanical Structure

As new materials, new structures and advanced control methods are rapidly developing, bionic robots are gradually moving from the stage of imitating the shape and behavioral gait of creatures to a biomimetic system based on electromechanical systems and biological characteristics. Bionic arm robots are mainly designed to imitate the human arm system and realize the characteristics and functions of human assignments. When compared with traditional mechanical arms, bionic arm robots are able to perform their assigned tasks efficiently under various complex task conditions in terms of high efficiency, low energy consumption and high environmental adaptability.

Bionic arm robots are mainly divided into bionic single-arm robots and bionic double-arm robots. It is not only possible for bionic two-armed robots to work together with both arms compared to anthropomorphic single-armed robots, but also are highly fault tolerant. This greatly improves the operational performance of bionic two-armed robots and their application ranges when they are confronted with complex tasks. Therefore, bionic dual-armed robots are urgently needed for the industrial, the medical, the educational and other fields [34].

**Figure 1:**
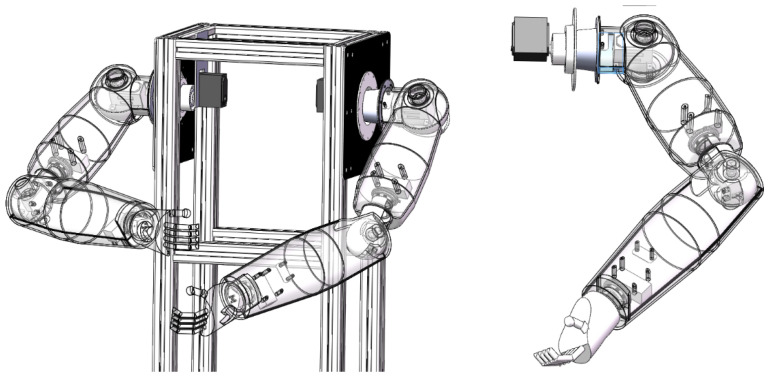
Mechanical structural design of anthropomorphic dual-arm robot.

The structural design of the anthropomorphic dual-arm robot is shown in Figure 1.

The anthropomorphic robotic arm is mainly divided into the performing parts and the functional parts, in which the performing part is equipped with five degrees of freedom. It is mainly realized with the functional part to move and rotate in a certain space. It is composed of two rotation degrees of freedom in the shoulder joint, two rotation degrees of freedom in the elbow joint and one rotation degree of freedom in the wrist. The functional part is mainly to realize the imitation of human hand movements. It is designed to grasp and release the objects. In this way, the anthropomorphic robotic arm will be able to accomplish grasping and releasing objects in a certain space through the cooperative operation between the executive part and the functional part. Therefore, anthropomorphic robotic arms are widely accepted in industrial and medical fields [35]. Other detailed parameters are shown in Table 1.

### 2.2. Acquisition System Components

The bionic dual-arm robots are mainly composed of multiple sensors to collect signals, such as Kinect and data gloves, and these signals are fused and processed for accurate control of the robots, in which the Kinect is used to collect the posture data of human arms and the data gloves are used to receive the signals of human hand movements. Their detailed functions and parameters are as follows.

The Kinect’s core technology is skeleton tracking technology, which is not only able to accurately identify the relative position relationships between human skeletal points in space, but also is capable of obtaining the 3D coordinate information about each joint of the human body through the depth image data processing algorithm. The Kinect-based skeleton tracking technology is able to convert the identified human skeletal data into human joint angle data, and intelligent algorithms can be used to map the collected joint data to each servo of the bi-arm robots, through the mutual cooperation of the servos, so as to achieve the precision control and fast response of the bi-arm robots.

**Figure 2:**
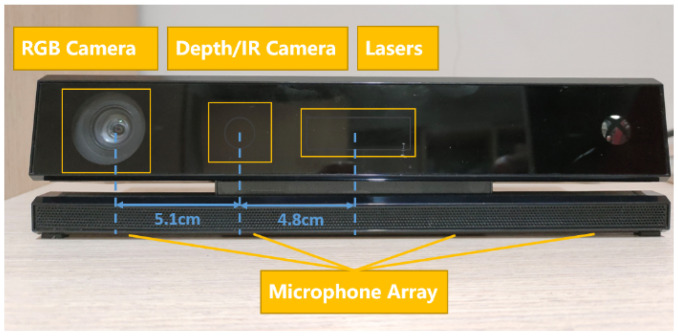
The primary functional components of Kinect.

The Kinect sensor is used to capture the operator’s arm joint position and angle information. The effective range of Kinect detection is 0.5 m–4.5 m, with a visual range of 70 degrees horizontally and 60 degrees vertically. It can track up to 6 users’ position information and support up to 25 skeletal nodes. As shown in Figure 2, the Kinect has two cameras: an RGB camera on the left to acquire 1920 × 1080 color images at up to 30 frames per second, a depth sensor in the middle to detect the relative position of the operator and an infrared emitter on the right to actively project modulated near-infrared light.

**Figure 3:**
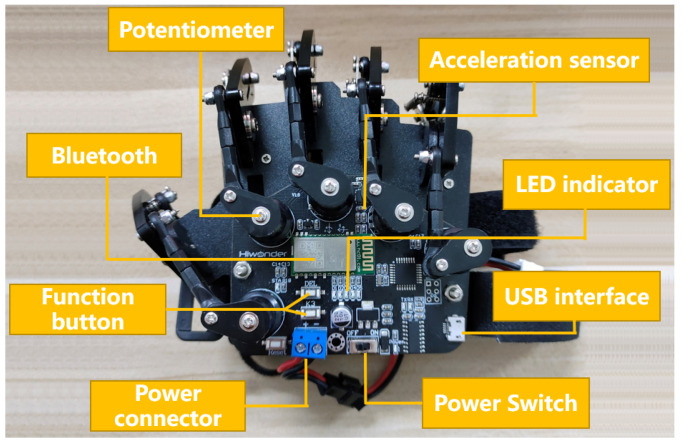
Data glove and its principal functional components.

The data glove collects information about the operator’s finger joint angle and palm rotation. Figure 3 shows the structure of the data glove. It has embedded Bluetooth HC-08 4.0BLE, and the master mode automatically connects to the BLE slave. It contains five potentiometers to detect the bending angle of each of the five fingers. It is equipped with a gyroscopic acceleration sensor to detect the angular velocity, acceleration and tilt information of the palm.

The control board type is Arduino UNO and the processor core is ATmega328. The control board has 14 digital inputs/outputs (6 of which can be used as PWM outputs), 6 analog inputs, a 16 MHz crystal oscillator, a USB port, a power socket, an ICSP header and a reset button in Figure 3.

The operator needs to initialize before using the glove: extend the arm and clench the fist, move the fist vertically down and turn on the power. The middle LED will flash rapidly several times, then the user extends the hand as far as possible. The LED flashes again, indicating that initialization is complete.

### 2.3. Data Preprocessing

Kalman filtering is a mathematical technique used for estimating the state of a system, given noisy and uncertain measurements. It is often used in robotics and control systems to improve the estimation and prediction of system states. The algorithm provides a way to optimally combine prior knowledge, current measurements and a model of the system’s dynamics to estimate the current state. In the context of a robotic arm following an experimenter’s arm motion, the Kalman filter can be used to estimate the position, velocity and acceleration of the experimenter’s arm using sensor measurements, such as data from cameras, inertial measurement units (IMUs) or other tracking devices. The measurements may be noisy, and there could be latency or delays in the processing pipeline. The Kalman filter helps in dealing with these uncertainties, providing a more accurate and timely estimate of the experimenter’s arm motion. By improving the estimation of the experimenter’s arm motion, the Kalman filter can help the robotic arm to react more quickly and accurately, reducing the delay time difference between them.

Based on the discrete-time linear stochastic difference equation, the state space model of the arm is

(1) $$x_{k}=Ax_{k-1}+Bu_{k-1}+w_{k-1}$$

(2) $$z_{k}=Hx_{k}+v_{k},$$

where $x_{k-1}$ is the arm state at the previous moment, $x_{k}$ is the estimated value of the arm state at the current moment and $z_{k}$ is the measured value of the arm state at the current moment. *A*, *B* and *H* are the state transition model, the control input model and the observation model, respectively. $w_{k-1}$ and $v_{k}$ are the process noise at the previous moment and the measurement noise, respectively, assuming that they are independent and obey a normal distribution *N*.

(3) $$\left.w_{k}\right.∼N\left(0,Q\right)$$

(4) $$\left.v_{k}\right.∼N\left(0,R\right)$$

where *Q* is the covariance of the process noise and *R* is the covariance of the measurement noise. For the case of hand motion analysis, the control input $u_{k}$ emitted in the muscle system is considered to be unknown. The motion model of the simplified arm is

(5) $$x_{k}=Ax_{k-1}+w_{k-1}.$$

The state of the arm position including the shoulder, elbow and wrist is

(6) $$x_{pos,k}=P_{k}$$

(7) $$x_{shoulder,k}=x_{shoulder,k}$$

(8) $$x_{elbows,k}=x_{elbows,k}$$

(9) $$x_{wrist,k}=x_{wrist,k}.$$

In Equation (6), $P_{k}$ is the position $P_{k}=\left(P_{x,k},P_{y,k},P_{z,k}\right)$. The state transfer model and observation model of the simplified arm are, respectively,

(10) A=diag(1,1,1)

(11) H=diag(1,1,1).

The Kalman filter eliminates the regularity error due to jitter and abrupt changes to estimate the arm (shoulder, elbow and wrist) position. The Kalman filter algorithm, a recursive predictive filtering algorithm, is used to estimate the state of the process and to minimize the estimation mean square error. The covariance matrix of the prior state estimate and the prior error in each cycle are

(12) $${\hat{x}}_{\bar{k}}=A_{k}{\hat{x}}_{k-1}$$

(13) $$P_{\bar{k}}=A_{k}P_{k-1}A_{k}^{T}+Q,$$

where ${\hat{x}}_{\bar{k}}$ is the covariance matrix of the prior state estimate. The prior estimate of *x* is obtained from the posterior estimate of the previous moment and the input information, and $P_{\bar{k}}$ is the prior mean squared error. Kalman gain is updated in each loop to correct the state estimate of the posterior.

(14) $$K_{k}=P_{\bar{k}}H_{k}^{T}/\left(H_{k}P_{\bar{k}}H_{k}^{T}+R\right)$$

(15) $${\hat{x}}_{k}={\hat{x}}_{\bar{k}}+K_{k}\left(z_{k}-H_{k}{\hat{x}}_{\bar{k}}\right)$$

where $K_{k}$ is Kalman gain and ${\hat{x}}_{k}$ is the posterior state estimate. The covariance matrix of the errors is updated.

(16) $$P_{k}=\left(I-K_{k}H_{k}\right)P_{\bar{k}}$$

(17) $$Q=\left[\begin{array}{ccc} \sigma_{x}^{2} & 0 & 0\\ 0 & \sigma_{y}^{2} & 0\\ 0 & 0 & \sigma_{z}^{2} \end{array}\right]$$

where $P_{k}$ is the posterior mean squared error and Q is the system noise covariance matrix.

### 2.4. Implementation

**Figure 4:**
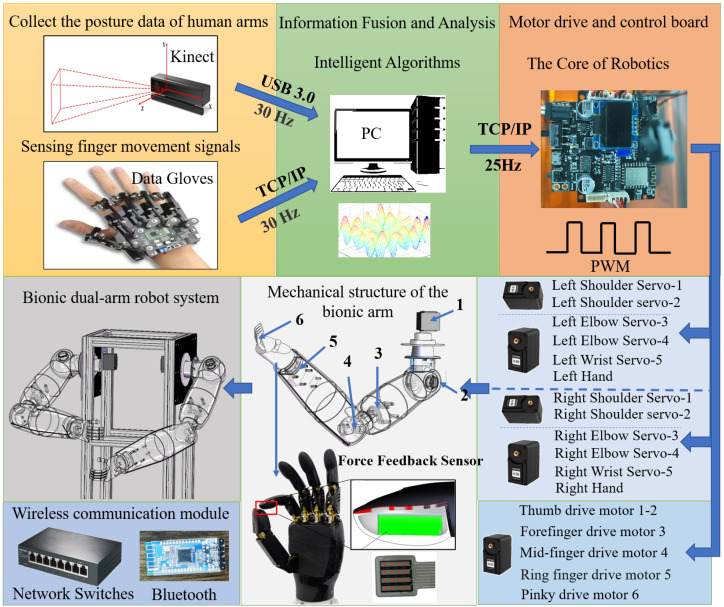
Bionic dual-arm robot system.

The proposed sensor fusion teleoperation control system is described as shown in Figure 4, which consists of three parts: the signal acquisition terminal, the control terminal and the communication protocol.

The acquisition terminal consists of an operator, Kinect devices and data gloves. The Kinect camera is used to collect the operator’s posture data of bilateral arms and data gloves are used to collect the operator’s posture data of bilateral hands.

The signal control terminal is a miniature control processor, which can communicate wirelessly through Bluetooth and TCP/IP. The control terminal is mainly composed of two parts: anthropomorphic robotic arm and bionic dexterous hand. The anthropomorphic robotic arm is used primarily as a mobile carrier. Each arm is servo-driven by 5 servos. There are 2 servos of 60 kg torque in the shoulder joint, 2 servos of 20 kg torque in the elbow joint and 1 servo of 20 kg torque in the wrist joint. Five servos are cooperated with each other to achieve the function of humanoid arm movement. Therefore, the anthropomorphic robotic arm is primarily used to reach the designated space location by carrying a dexterous hand.

**Figure 5:**
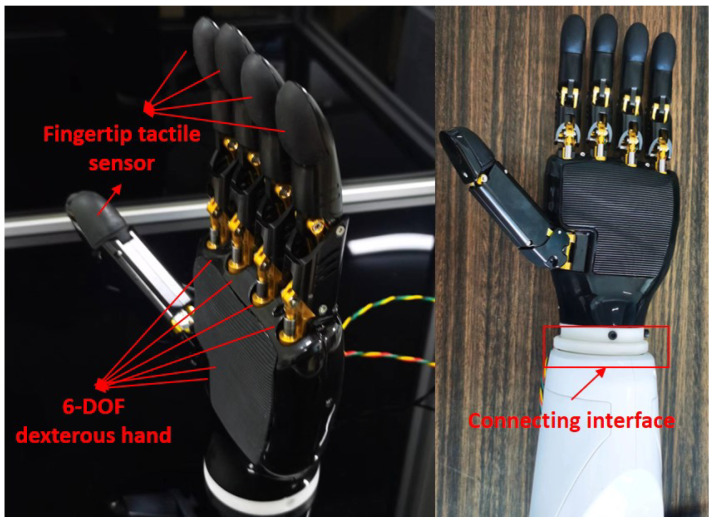
The display and installation of the dexterous hand.

The dexterous hand is consisted mainly of the hand mechanical structure, 6 driving servos and 5 finger force feedback sensors, as well as soft and hard connection interfaces, etc. Figure 5 is the display and installation of the dexterous hand. We can realize the pressure signal collection at the finger tips of the dexterous hand by setting the force feedback sensors, and further process the signal through our intelligent algorithm, so that the dexterous hand will be more accurate in perceiving the pressure changes of the external environment. Therefore, we are controlling the grip strength of the dexterous hand by using force feedback, which makes the dexterous hand have improved human–computer interaction performance. At the same time, the dexterous hand is not only able to achieve adaptive grasping according to the shape of the object, but also to implement some basic functions that imitate human hands, such as opening doors and carrying boxes.

In the teleoperation communication protocol, the acquisition terminal collects the operator’s bi-arm and bi-hand posture information. Through the MATLAB filtering processing and data fusion, the control algorithm is transmitted to the micro control processor of the dual-arm robots by means of Wi-Fi connection, so as to be driven by each servo for effective and precise control. Similarly, it is possible to give feedback on the positions to MATLAB through the microcontroller. Therefore, the calibration of the actual trajectory data of the multi-arm robots with the theoretical control data will be completed, and then the intelligent algorithm will be used to realize the control stability and reliability from the multi-arm robots. The communication connection between the MATLAB PC and the micro control processor is established in the same Wi-Fi environment, and the data are transmitted through the TCP/IP protocol.

## 3. Kinematic Modeling

Although bionic single-arm robots are widely used in industrial, medical and service fields, there are many limitations in the practical operation and functional realization of bionic single-arm robots, which greatly limit the collaborative operation of bionic single-arm robots in conditions such as equipment assembly or heavy lifting. In contrast, bionic two-armed robots are capable of accomplishing complex tasks that bionic single-armed robots are not able to achieve through the collaborative cooperation of both arms. Therefore, bionic two-armed robots have become the focus of research in the field of intelligent robotics.

### 3.1. D(Denavit)–H(Hartenberg) Modeling

**Figure 6:**
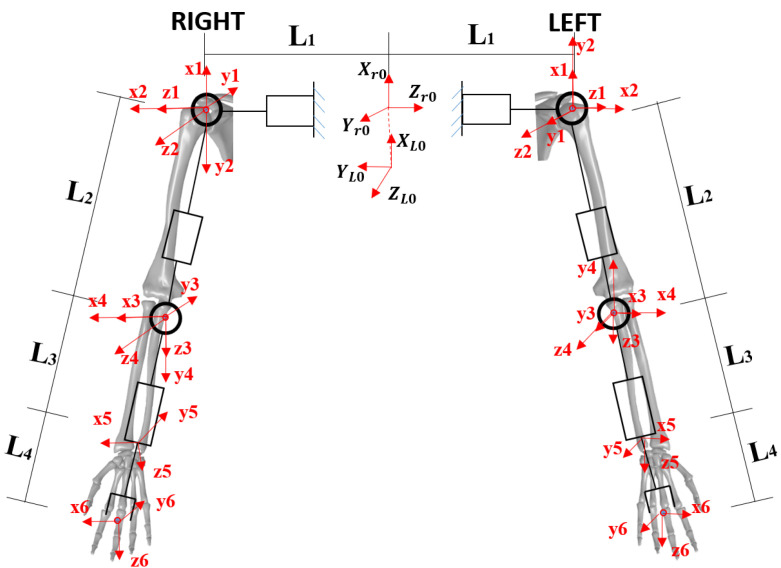
The double-arm anthropomorphic robot is constructed based on the improved DH method.

Firstly, we are required to number the connecting rods of the arm, we usually are defining the fixed base as connecting rod 0, the first movable connecting rod as connecting rod 1, and so on, and the end connecting rod of the arm is defined as connecting rod i. The modified DH method is to establish the transformation coordinate system i on connecting rod i, and it is a common rule to define the coordinate system of the connecting rod. The coordinate system of the 10-degree-of-freedom dual-arm system is established according to the modified DH method in Table 2, as shown in Figure 6.

The modified DH method is the XZ transformation process. The joint angle θ represents the rotation angle between coordinates with $Z_{i}$ axis as the rotation axis; the linkage offset d represents the distance along the $Z_{i}$ axis direction; the linkage twist angle α represents the rotation angle with $X_{i-1}$ axis as the rotation axis; the linkage length a represents the distance along the $X_{i-1}$ axis direction. The modified DH kinematic modeling is used and is formulated as follows.

(18) $${}_{i-1}^{i}T=Rot_{X_{i}}\left(\alpha_{i-1}\right)Trans_{X_{i}}\left(a_{i-1}\right)Rot_{Z_{i-1}}\left(\theta_{i}\right)Trans_{Z_{i-1}}\left(d_{i}\right)\;=\left[\begin{array}{cccc} 1 & 0 & 0 & 0\\ 0 & {cos\;\alpha }_{i-1} & -sin\;\alpha_{i-1} & 0\\ 0 & sin\;\alpha_{i-1} & cos\;\alpha_{i-1} & 0\\ 0 & 0 & 0 & 1 \end{array}\right]\cdot \left[\begin{array}{cccc} 1 & 0 & 0 & a_{i-1}\\ 0 & 1 & 0 & 0\\ 0 & 0 & 1 & 0\\ 0 & 0 & 0 & 1 \end{array}\right]\cdot \left[\begin{array}{cccc} {cos\;\theta }_{i}\; & -sin\;\theta_{i} & 0 & 0\\ sin\;\theta_{i} & {cos\;\theta }_{i} & 0 & 0\\ 0 & 0 & 1 & 0\\ 0 & 0 & 0 & 1 \end{array}\right]\cdot \;\left[\begin{array}{cccc} 1 & 0 & 0 & 0\\ 0 & 1 & 0 & 0\\ 0 & 0 & 1 & d_{i}\\ 0 & 0 & 0 & 1 \end{array}\right]$$

(19) $${}_{i-1}^{i}T=\left[\begin{array}{cccc} {cos\;\theta }_{i}\; & -sin\;\theta_{i} & 0 & a_{i-1}\\ sin\;\theta_{i}{cos\;\alpha }_{i-1} & cos\;\theta_{i}{cos\;\alpha }_{i-1} & {-sin\;\alpha }_{i-1} & {-d_{i}sin\;\alpha }_{i-1}\\ sin\;\theta_{i}{sin\;\alpha }_{i-1} & cos\;\theta_{i}{sin\;\alpha }_{i-1} & {cos\;\alpha }_{i-1}\; & {d_{i}cos\;\alpha }_{i-1}\\ 0 & 0 & 0 & 1 \end{array}\right]$$

In this formula, *i* is the link, when *i* = 0 it indicates the base and i=1 is the first link. ${}_{i-1}^{i}T$ is the homogeneous transfer matrix of the *i* link relative to the i−1 link. The equation is obtained after superposition as follows:

(20) $$T={T_{1}^{0}T_{2}^{1}T_{3}^{2}\ldots T}_{i}^{i-1}$$

### 3.2. Based on MATLAB Modeling

**Figure 7:**
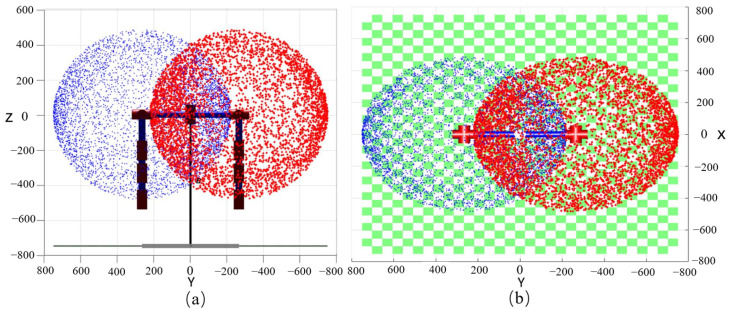
The maximum working range of the anthropomorphic double-arm robots. (**a**) YZ plane view; (**b**) XY plane view.

The mathematical model of the five-degrees-of-freedom humanoid robot arm is obtained by using the MATLAB toolbox. As shown in Figure 7, the maximum working range of the anthropomorphic double-arm robots was reached in the three-dimensional space. Firstly, the kinematic model of the anthropomorphic double-armed robots was established in the MATLAB toolbox through the modified DH parameters, while the point cloud map of the maximum range reached by the anthropomorphic two-armed robot in the three-dimensional working space was established by using the Monte Carlo method. The Monte Carlo method, also known as the statistical simulation method, is a numerical computation method guided by probabilistic statistical theory. The advantage of using the Monte Carlo method to calculate the workspace is the short time taken compared to numerical.

The steps of Monte Carlo method workspace solving are as follows:

(1) Random variables are generated for each joint, and a set of joint space vectors for the robotic arm are generated at random.

(2) The calculation of the kinematic positive solution, and the mapping from the joint space to the end workspace (Cartesian coordinate system) is performed.

(3) The workspace distribution is plotted.

### 3.3. Control Model

The recognized human joint angles are converted into servo control commands according to the algorithm for bi-arm robot motion control. The shoulder of the bimanual robots was controlled individually by two servos to drive two degrees of rotation freedom in the shoulder. Similarly, the elbow joint was equipped with two degrees of rotational freedom, and the wrist joint was provided with only one degree of rotation freedom along the axis direction. Therefore, the Kinect is acquiring the angle information of the human back arm movement in 3D space, and the intelligent algorithm is completing the conversion process between the angle information and the servo commands. Its principle is as follows:

(1) Shoulder joint: The number 1 degree of freedom of the robot arm is controlled in real-time by the value of the angle variable between the projection line of the upper arm in the side plane from the human body and the front plane, and the number 2 degree of freedom of the robot arm is coordinated in real-time by the angle variable between the projection line of the upper arm in the front plane from the human body and the lateral plane. Then, the two-angle information is mapped to the two servos in the shoulder joint of the bimanual robot, and therefore the rotation angle that the two servos of the shoulder joint need to achieve is determined. Meanwhile, the angle-command conversion algorithm is used to obtain the precise command of the servos. Thus the accurate control of the shoulder joint is completed.

As shown below, Equation (21) is expressed as the projection of the line on the plane, and Equation (22) is expressed as the angle between the line and the plane, so that the angle of the spatial position of the 2 degrees of freedom of the shoulder can be accurately calculated to accurately control the two-armed robot.

(21) $$\vec{a_{p}}=\vec{a}-\vec{a^{\prime }}=\vec{a}-\frac{\vec{a}\cdot \vec{n}}{∥\vec{n}{∥}^{2}}\vec{n}$$

(22) $$\phi =\arcsin\left(\frac{|\vec{n}\cdot \vec{d}|}{|\vec{n}\left|\right|\vec{d}|}\right),\phi \in \left[0^{\circ },90^{\circ }\right]$$

**Figure 8:**
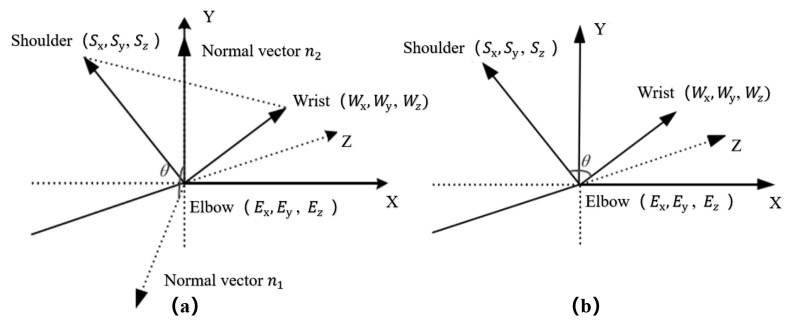
Schematic diagram of mathematical model of human elbow joint. (**a**) The angle control model analysis of the number 3 degree of freedom; (**b**) The angle control model analysis of the number 4 degree of freedom.

(2) Elbow joint: As shown in Figure 8, the number 3 degree of freedom of the elbow joint is controlled in real time by the dynamic change of the angle between the plane formed by the three points of shoulder–elbow–wrist and the ZOX plane; the number 4 degree of freedom of the elbow joint is controlled in real time by the dynamic change of the angle between the two vectors of elbow–wrist and elbow–shoulder.

(3) Wrist joint: the number 5 degree of freedom of the wrist joint is precisely controlled by the inertial sensor of the glove.

In this way, it is used by image acquisition technology, finger motion sensing technology and the intelligent control algorithm. Thus it accomplishes real-time control of the bionic dual-arm robots and achieves complex collaborative operations.

**Figure 9:**
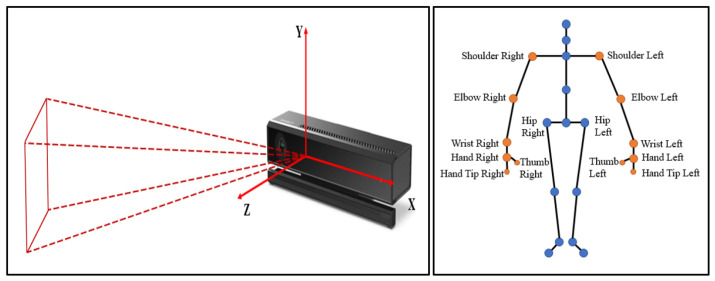
The area of the Kinect camera for capturing images (**left**); The skeleton points of the human body are recognized by the Kinect system (**right**).

There is an absolute coordinate system established on the Kinect and a relative coordinate system was determined on the operator’s shoulder. The absolute coordinate system was defined as a spatial coordinate system with the Kinect’s depth camera as the origin. The human skeletal points were recognized by Kinect, as shown in Figure 9.

## 4. Experiments

### 4.1. Human–Computer Interaction Experiments

#### 4.1.1. Experiment with Single-Handed Control

**Figure 10:**
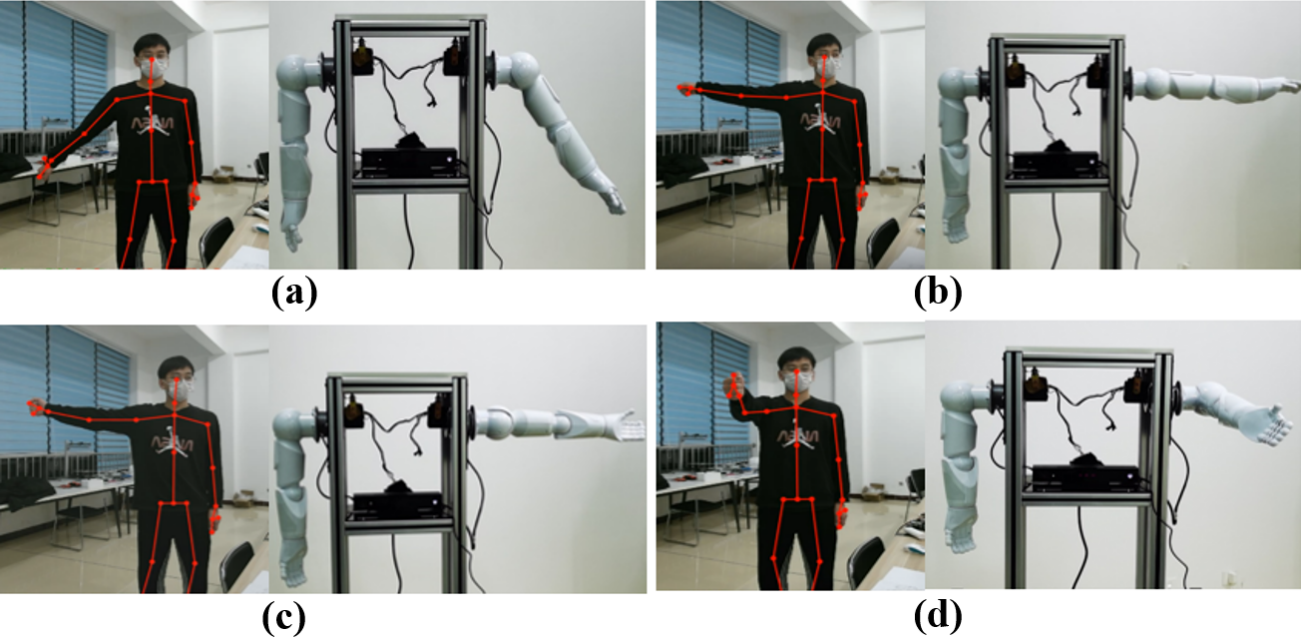
Experiment with Single Handed Control. (**a**) Single-arm shoulder joint swing action; (**b**) Single-arm shoulder joint horizontal extension; (**c**) Turned arm, palm up; (**d**) Single-arm horizontal forward flexion.

The single-arm controlled experiment process is shown in Figure 10. Firstly, the experimental equipment was energized, and running data acquisition was performed by the control software platform. The experimenter was standing in front of the Kinect body tracker at a distance of about 2 m. On this computer screen real-time displayed the experimenter’s arm posture and the coordinates of the skeletal points. Meanwhile, the data collected were transferred to the data processing system to accomplish the data collection. Then, the experimenter completed, at a certain speed (Figure 10a–d), the four posture states. Furthermore, the experimenter was asked to gradually increase the motion speed to complete these four poses. The three groups of experimental data are collected and analyzed. Finally, the design called for different experimenters to conduct the same experiment as above, and it was concluded that, as the experimenter’s arm motion speed increased, the interactive motion of the bionic arm with the delay time difference gradually increased, and the delay time difference is basically the same regardless of which experimenter’s laboratory was used.

We applied a combination of jitter-cancellation filtering algorithm and Kalman filtering algorithm while conducting three sets of experiments with different motion speeds for each experimenter again, and we analyzed the experimental data and found that the combination of jitter-cancellation filtering algorithm and Kalman filtering algorithm greatly improved the delay time difference between the bionic arm robot and the experimenter’s arm motion. This is because the use of the Kalman filter algorithm can reduce the delay time difference by predicting and correcting the measured data. The algorithm estimates the actual state of the human motion and predicts the next state based on the previous state and measurements. When transmitting the data, the algorithm corrects for the predicted values, thus reducing the delay time difference and improving the real-time and accuracy of motion control. Thus, the application of the jitter cancellation filter and Kalman filter algorithm significantly improves the delay time difference between the robot and the experimenter’s arm motion.

#### 4.1.2. Experiment with Double Hand Control

**Figure 11:**
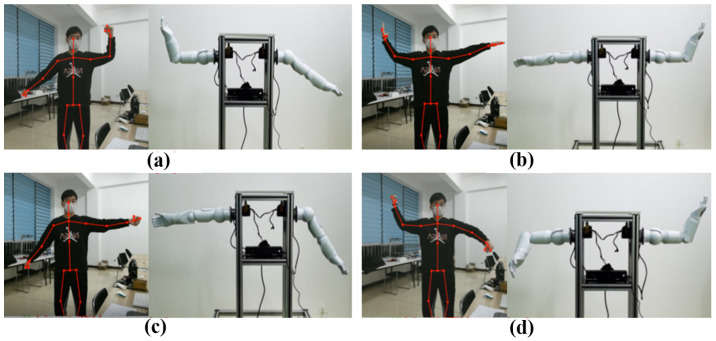
Experiment with double hand control. (**a**) Left arm raised, right arm swings sideways; (**b**) Left arm extended horizontally, right arm raised; (**c**) Left arm extended horizontally, right arm swings sideways; (**d**) Left forearm flexed down, right arm raised.

Based on the anthropomorphic dual-arm manipulator control system, it was tested for six experimenters in the same indoor environment. The results show that the dual-arm robot control system can accurately perceive the posture data of the operators’ arm and hand motions and imitate the movements of human arms in real-time to achieve anthropomorphic functions. Therefore, the anthropomorphic robot arm teleoperation control based on sensor fusion is broadly applicable and more flexible and convenient to be used. The integration and control of multimodal signals are more bionic in nature and more in compliance with the characteristics of bionics, and they are able to improve the coordination and naturalness of the dual-arm robot control. As shown in Figure 11, the anthropomorphic robotic arm is mainly supplied with data through the Kinect collection of human arm movement data and the data glove sensing human hand motion information. At the same time, combined with force feedback control data, multi-sensor information fusion and control are achieved. Sensor-fused teleoperated control is more efficient and stable than unimodal control strategies, as shown in Figure 10.

#### 4.1.3. Experiments in Dexterous Hand Control

**Figure 12:**
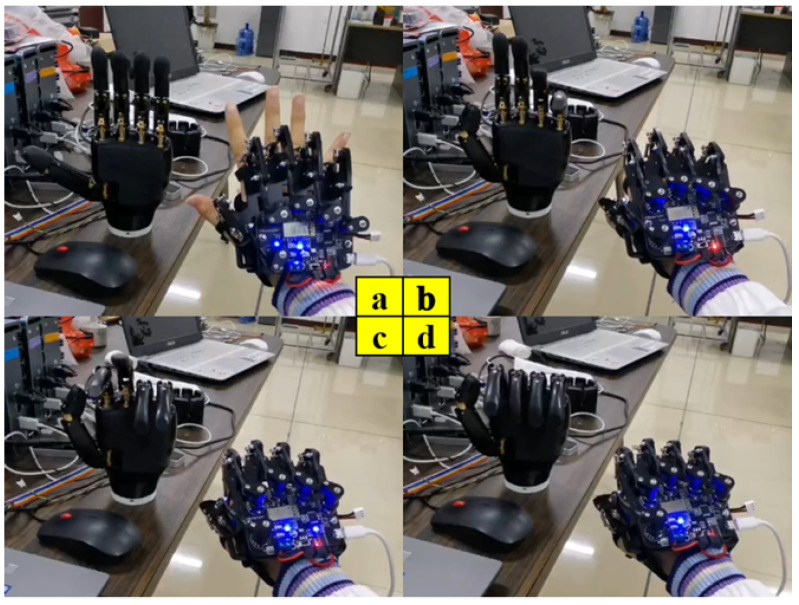
Data gloves control dexterous hands in real time. (**a**) Five fingers open; (**b**) Thumb movement; (**c**) Controlled contact between index finger and thumb; (**d**) Clenching of the fist.

The anthropomorphic robot arm is equipped with the dexterous hand in order to perform functions such as object transfer and humanoid hand movements, so it is crucial for the dexterous hand to be used. The data glove is equipped to control the dexterous hand by using five potentiometers to sense changes in finger movement in real-time, therefore outputting the corresponding voltage value signal. Thus, we take advantage of the data glove to sense the motion posture of the human hand, so that the signals of these hand joints are processed by data fusion and Kalman filtering, and the motion signals of each human finger joint are mapped to the driving servos of the dexterous hand in a stable manner. As shown in Figure 12, the data glove controls the dexterous hand in real time. At the same time, the feedback signal of the fingertip force is felt during the movement of the dexterous hand. We fuse and analyze the perceived data through an intelligent algorithm, so as to realize the control mode of combining human hand motion control and force feedback control. The data of multiple sensors can achieve the constant force control and adaptive control mode of the dexterous hand through the intelligent algorithm. This greatly expands the adaptability of the dexterous hand to the environment and application scenarios compared to the dexterous hand without force feedback.

**Figure 13:**
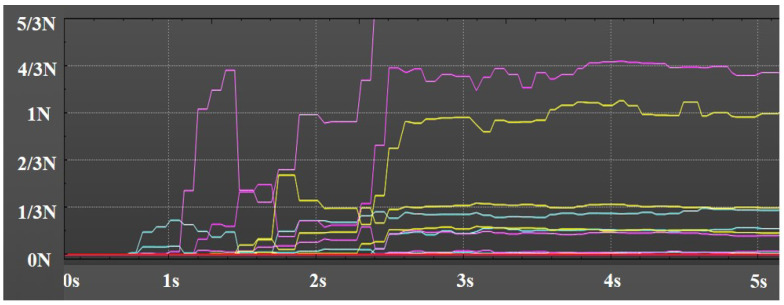
Pressure feedback from the 16 contacts of the tactile sensor on the tip of the index finger when grasping the bottle.

Figure 13 displays pressure feedback from the 16 contacts of the tactile sensor on the tip of the index finger when grasping the bottle. At the beginning of grasping the bottle, the contact pressure is 0. When the maximum pressure among the 16 contacts reaches 1 N, the manipulator cannot be controlled and the grasp is successful. When the maximum pressure is less than 0.5 N, the manipulator can be controlled and released successfully.

### 4.2. Information Collection and Processing

Since the placement of the Kinect sensor is affected by the shape of the subject, the placement of the Kinect will influence the generated image. For example, when the target (e.g., a person) is standing straight in the range of the Kinect sensor, but the Kinect sensor itself is tilted, the resulting image will be skewed. The experimental process was performed with the subject standing about 2 m in front of the Kinect and facing the Kinect camera sensor, and then it was kept in a static motion on the right arm extension. Utilizing the Kinect camera to perceive the skeletal point data of the human body in 3D space in real-time, the data of real-time perceived human motion will produce a jitter phenomenon over time, which will in turn cause the accumulation of control errors. Therefore, the 3D-coordinate information is collected from the shoulder joint, elbow joint and wrist joint of the human arm, and the changing conditions of the date are calculated respectively in the three directions of x, y and z. Meanwhile, we collected the information of the human arm movement and performed Kalman filtering. The intelligent filtering algorithm is mainly used to eliminate the regular errors generated in the experimental process.

**Figure 14:**
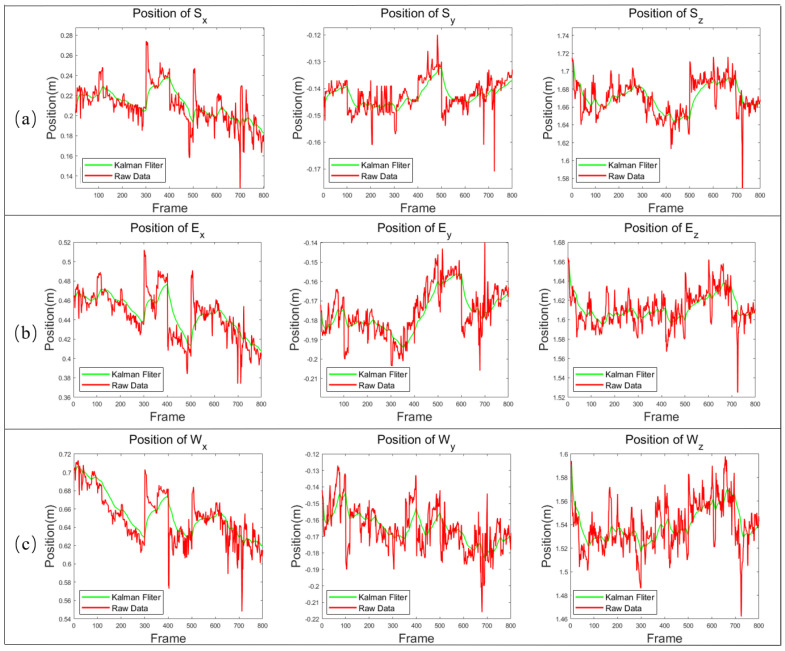
Data acquisition and filtering processing of joint position in x, y and z directions respectively. (**a**) Shoulder joint S; (**b**) Elbow joint E; (**c**) Wrist joint W.

The experimental data in this paper are significantly spiked, and the jitter elimination filtering algorithm is used to suppress the spikes in the input by limiting the allowed changes in the output of each frame. Therefore, the Kalman filtering algorithm is used to improve the data jitter. The data of 800 frames are taken for analysis and filtered here, as shown in Figure 14.

**Figure 15:**
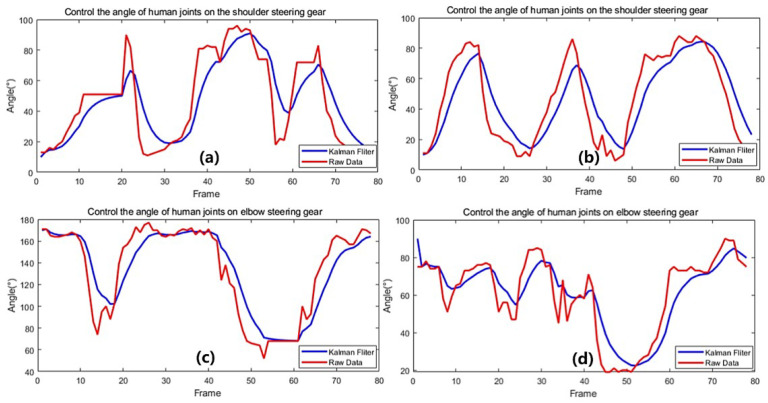
Real-time acquisition and Kalman filtering of shoulder and elbow joint angles. (**a**) Acquisition and filtering of the shoulder joint 1 angle data; (**b**) Acquisition and filtering of the shoulder joint 2 angle data; (**c**) Acquisition and filtering of the elbow joint 1 angle data; (**d**) Acquisition and filtering of the elbow joint 2 angle data.

Firstly, we collected the motion jitter information of human arm in 3D space and Kalman filtered the collected data in order to eliminate the larger jitter errors. Then, we calculated the movement angles of the shoulder and elbow joints with the filtered data and the above calculation method, while the angle changes of the shoulder and elbow joints were Kalman filtered again to obtain the data as shown in Figure 15, namely, real-time acquisition and Kalman filtering of shoulder and elbow joint angles. This is achieved by the fusion of two Kalman filters, which makes the process of controlling the motion of the anthropomorphic robotic arm more stable and smooth.

## 5. Conclusions

The humanoid dual-arm robots are designed and controlled from the perspective of bionics in this paper, and the Kinect depth camera and data glove are used to collect the operator’s dual-arm posture data. This solves the shortcomings of single modal signal control robots. Meanwhile, the fused data are analyzed and processed by fusing the body-sensing glove data, and the intelligent control algorithm is added to achieve a more stable and accurate anthropomorphic dual-arm robot tracking and control. This not only implements remote operation control of the humanoid robot but also effectively strengthens the naturalness and flexibility of the control. Therefore, anthropomorphic dual-arm robots are capable of collaborative operation and environmental adaptation under complex environmental conditions and will be widely used in industrial, medical and educational fields because of their high operational efficiency and low energy consumption, and high flexibility.

Although we achieved promising control performance using the sensor fusion technology, the time delay is still not fully addressed and discussed in this study, which is of vital importance for teleoperation control. It is believed that in future work, we will fuse multiple vision sensors and myoelectric sensors and we intend to use multi-sensor fusion algorithms and intelligent control algorithms, which further improve the control accuracy and coordination. At the same time, the control performance of time delay will be discussed and addressed to improve transparency.

## Figures and Tables

**Table 1:** Mechanical structure parameters.

Technical Requirements		Parameters
Degree of freedom for anthropomorphic arms		Five degrees of freedom (single arm)
The drive form of anthropomorphic robotic arm		Servo direct drive
Mechanical arm load capacity (number)		20 kg (3), 60 kg (2)
Geometric dimensions	Shoulder positioning	115 mm
	Upper Arms	205 mm
	Forearm	130 mm
	Anthropomorphic hand	200 mm
Maximum range	Shoulder 1 Servo	0–360°
	Shoulder 2 Servo	0–180°
	Elbow 3 Servo	0–180°
	Elbow 4 Servo	0–90°
	Wrist 5 Servo	0–180°
Degree of freedom for the dexterous hand		Six degrees of freedom (single-handed)
Fingertip force feedback sensor		Multi-array flexible tactile sensors
Basic parameters of force sensors	Area	15 mm ∗ 15 mm
	Array Points	16 (4 ∗ 4)
	Force measurement accuracy	±0.2 N

**Table 2:** Modified DH Parameters of the anthropomorphic bi-arm robot.

Link (i)	θ (rad)	d (mm)	a (mm)	α (rad)	Offset (rad)
Left Shoulder Roll link	$\theta_{1}^{L}$	${Ł}_{1}$ (265 mm)	0	pi/2	pi/2
Left Shoulder Pitch link	$\theta_{2}^{L}$	0	0	−pi/2	−pi/2
Left Elbow Roll link	$\theta_{3}^{L}$	${Ł}_{2}$ (205 mm)	0	pi/2	0
Left Elbow Pitch link	$\theta_{4}^{L}$	0	0	−pi/2	0
Left Wrist Roll Link	$\theta_{5}^{L}$	${Ł}_{3}$ (130 mm)	0	pi/2	0
Left Hand	0	${Ł}_{4}$ (150 mm)	0	0	0
Right Shoulder Roll link	$\theta_{1}^{R}$	${Ł}_{1}$ (265 mm)	0	−pi/2	pi/2
Right Shoulder Pitch link	$\theta_{2}^{R}$	0	0	pi/2	−pi/2
Right Elbow Roll Link	$\theta_{3}^{R}$	${Ł}_{2}$ (205 mm)	0	−pi/2	0
Right Elbow Pitch Link	$\theta_{4}^{R}$	0	0	pi/2	0
Right Wrist Roll Link	$\theta_{5}^{R}$	${Ł}_{3}$ (130 mm)	0	−pi/2	0
Right Hand	0	${Ł}_{4}$ (150 mm)	0	0	0
